# Effects of Mesobiliverdin IXα-Enriched Microalgae Feed on Gut Health and Microbiota of Broilers

**DOI:** 10.3389/fvets.2020.586813

**Published:** 2021-01-20

**Authors:** Cheng-Wei T. Chang, Jon Y. Takemoto, Pei-En Chang, Madher N. AlFindee, Yuan-Yu Lin

**Affiliations:** ^1^Department of Chemistry and Biochemistry, Utah State University, Logan, UT, United States; ^2^Department of Biology, Utah State University, Logan, UT, United States; ^3^Institute of Biotechnology, National Taiwan University, Taipei City, Taiwan; ^4^Department of Animal Science and Technology, National Taiwan University, Taipei City, Taiwan

**Keywords:** gut health, microbiota, mesobiliverdin, spirulina, poultry

## Abstract

Gut inflammatory bowel diseases (IBDs) links to animal medicinal feed and antibiotic-resistance are fueling major economic impacts in the agricultural livestock industry. New animal feeds that promote livestock gut health and control of IBDs without antibiotics are needed. This study investigates the effects of mesobiliverdin IXα (MBV)-enriched microalgae spirulina extracts on the growth performance, blood parameters, intestinal morphology, and gut microbiota of broilers. A total of 288 1-day-old broiler chicks (Arbor Acres) were randomly allotted to six dietary treatments (4 pens/treatment and 12 birds/pen). The dietary treatments comprised a basal diet as control (CON), basal diet plus 0.05 and 0.1% microalgae extract as low and high dose, respectively (SP1 and SP2), basal diet plus 0.05 and 0.1% MBV-enriched microalgae extract as low and high dose, respectively (MBV-SP1 and MBV-SP2), and basal diet plus 0.1% amoxicillin (AMX). All treated animals showed no significant differences in live weight, average daily gain, and feed efficiency compared to control animals. Histological examination showed that AMX treatment decreased the villi lengths of the duodenum and ileum below control villi length (*P* < 0.05) while MBV-SP1 and particularly MBV-SP2 increased villi lengths in the duodenum, jejunum, and ileum above AMX -treatment lengths (*P* < 0.05). The *Firmicutes*/*Bacteroidetes* ratio increased in the cecum of broilers fed AMX (*P* < 0.05) while SP2, MBV-SP1, and MBV-SP2-fed animals showed (in order) increasing ratios up to the AMX level. The abundance of bacterial species of the genus *Lactobacillus* increased in MBV-SP1 and MBV-SP2-fed groups including a striking increase in *Lactobacillus salivarius* abundance with MBV-SP2 (*P* < 0.05). Feeding MBV-SP1 and MBV-SP2 decreased the level of pro-inflammatory cytokine IL-6 in plasma of broilers to a greater extent than SP1 and SP2. These results reveal that MBV-enriched microalgae extracts improve the intestinal health and beneficial microflora composition of broilers.

## Introduction

Animal illnesses are globally widespread and challenging clinically, scientifically, and socio-economically (1). Well-known in human diseases, inflammatory bowel diseases (IBDs) are also prevalent and increasingly serious diseases for agricultural livestock. Johne's disease in cattle and other ruminants (2), antibiotic-associated colitis in pigs and horses (3), and necrotic enteritis in chickens (4) are examples of livestock diseases having large impacts on global agro-economies and food security. Complex interactions occur between the gut microbiota and intestinal immune defense systems. Imbalances and disruption of the normal interactions between these systems result in inflammatory responses that characterize IBDs (1, 2, 4, 5). A role for inflammation in IBDs points to anti-inflammatory therapies to combat these diseases (6). However, the majority of anti- inflammatory approaches against IBD either lack effectiveness, are high cost to produce, or have undesirable side effects. For example, a once promising anti-TNFα therapy is expensive and shows loss of response and undesirable side-effects (7). Natural product anti-inflammatories such as curcumin are possibilities though poor bioavailability following ingestion is a limitation (8).

Spirulina microalgae (mainly cyanobacteria *Arthrospira platensis and Arthrospira maxima*) are grown commercially in large quantities (nearly 3,000 tons per year) for food and feed by many companies worldwide (9). Spirulina not only has potential as a sustainable biofuel, but also as an animal feed (10). Nutrients of spirulina enrichening with protein, carbohydrates, balanced amino acids, carotenoids, fatty acids including γ-linolenic acid, vitamins, and minerals (11, 12). Approximately half of the total spirulina production is currently used for livestock and fish feed. The agricultural use of spirulina as a feedstock is anticipated to gradually grow (9, 13). Furthermore, spirulina has the beneficial effects of anti-oxidation, immunomodulation, and microbial-modulating activities in chickens and pigs, and also good potential for enhancing antibiotic effects (13–15). The cellular protein contents of spirulina and other microalgae are relatively high, and numerous experimental trials with agricultural animals support good productivity, animal health, and product quality with microalgae-supplemented feed (16, 17).

In animals, bioactive tetrapyrrole metabolites are derived by ring cleavage of heme by the enzyme heme oxygenase-1(HO-1). Biliverdin IXα, carbon monoxide, and iron are the initial products from HO-1 action. Biliverdin IXα is subsequently reduced via biliverdin reductase to bilirubin that is excreted in bile. The overall process serves to eliminate heme–which is toxic when accumulated. Biliverdin IXα is also produced by microbes and plants. In microalgae (cyanobacteria and red algae), biliverdin IXα is a precursor to photosensitive tetrapyrroles such as phycocyanobilin and phycoerythrobilin which are chromophores for microalgal light-harvesting systems. Biliverdin IXα, bilirubin and phycocyanobilin are powerful antioxidants (18) and biliverdin IXα and bilirubin are effective cytoprotectants against oxidative stress (19). Biliverdin IXα activates biliverdin reductase to signal downstream pathways for anti-inflammatory cytokine production and activities and suppression of pro-inflammatory gene expression (20). Biliverdin IXα administration has been shown to suppress or protect against several acute and chronic inflammatory diseases such as colitis (21), gastroenteritis (22), sepsis-induced intestinal inflammation and dysmotility (23), and intestinal ischemic/reperfusion injury (24). Thus, biliverdin IXα-based therapies have been suggested for treating human IBD diseases (21, 22).

Though biliverdin IXα as a therapeutic has been considered for over 10 years (19), its clinical use is hampered by insufficient quantity, uncertain purity, and derivation from mammalian materials. As an alternative, mesobiliverdin IXα (MBV), a close biliverdin IXα analog, was synthesized from microalgae phycocyanin with high purity and in large amounts by our research group (25, 26). MBV differs from biliverdin IXα with ethyl groups in place of vinyl groups at positions 3 and 18 of the tetrapyrrole structure. MBV and biliverdin IXα are equally good substrates for human BVR, and they impart similar degrees of cytoprotection against oxidative stress in pancreatic islets (27). MBV thus appears to possess therapeutic potential similar to that of biliverdin IXα with the added benefits of scalable production in large amounts from a non-animal source. However, there is no relevant study on the use of MBV in animal feed to promote animal health. The research proposed here will further test these notions in addition to the main focus of exploring MBV's possible beneficial effects in microalgae-based animal feed.

## Materials and Methods

### Animal, Feed, and Dietary Treatments

Two hundred and eighty-eight Arbor Acres broilers were reared from day 1 to day 30 and randomly allotted to 6 dietary treatments, 4 replicates for each treatment, and 12 chicks per replicate. Chickens were fed isonitrogenous and isocaloric corn-soybean meal based on calculated values of the ingredients to meet the recommendations for broilers (27). The birds were reared in floor pens (108 × 52 cm) and bedded with rice hulls. The chickens were illuminated at night. Electric incandescent light was used to keep chicks warm. All chicks were kept at 20 lux of light intensity on 23L:1D photoperiod for the week 1. At day 8, the broilers were exposed to the same light intensity and maintain 17L:7D photoperiod thereafter (28). The basal diet composition in the starter and finisher are shown in Table 1. The diets were in a mash form and provided *ad libitum*, and the birds had free access to tap water. For dietary treatments, basal diet feed was supplemented with amoxicillin (AMX) (antibiotic growth promoter, 0.1% by weight), Spirulina microalgae extracts SP1 and SP2 (0.05 and 0.1%, respectively by weight), or MBV-enriched microalgae extracts MBV-SP1 and MBV-SP2 (0.05 and 0.1%, respectively, by weight). Microalgae extracts for making SP1 and SP2 were prepared as described previously (25) from Spirulina powder (*Arthrospira platensis*), purchased from Bio-Alternatives, 834 Richmond Street, Klamath Falls, OR 97601 RMac Enterprises Inc. (https://www.bio-alternatives.net/). MBV-enriched microalgae extracts MBV-SP1 and MBV-SP2 were prepared by exposure of microalgae extracts to metal salts as previously described (26). From high-performance liquid chromatography analyses (25), MBV-SP1 and MBV-SP2 extracts were estimated to contain between 0.28 and 0.84 mg of MBV per g of dry microalgae extract, respectively. These levels of MBV-supplementation to animal feed were previously observed to suppress colitis in mouse model feeding studies (YY Lin, unpublished observations).

**Table 1:** Basal diet composition and nutrient content.

	**Day 1–21**	**Day 22–30**
Ingredient (as fed-basis %)		
Yellow corn	51.22	58.80
Soybean meal	40.00	33.00
Soybean oil	4.5	4.49
Choline	0.15	0.15
BHT^a^	0.02	0.02
Dicalcium phosphate	1.65	1.21
Calcium carbonate	1.63	1.60
DL-methionine	0.18	0.08
NaCl	0.35	0.35
Vitamin premix^b^	0.15	0.15
Mineral premix^c^	0.15	0.15
Total	100	100
Nutrient (calculated)		
ME^d^ (kcal/kg)	3340.31	3384.34
Crude protein (%)	23.16	20.41
Total lipid (%)	6.99	7.16
Lysine (%)	1.33	1.14
Methionine (%)	0.60	0.39
Methionine + cysteine (%)	0.95	0.78
Calcium (%)	1.00	0.91
Non-phytate phosphorus (%)	0.45	0.36

a *BHT, butylated hydroxytoluene*.

b *Provided per kg of diet (vitamin): vitamin A, 10,000 IU; vitamin D_3_, 2,000 IU; vitamin E, 15 mg; vitamin K, 4 mg; thiamine, 2 mg; riboflavin, 6 mg; pyridoxine, 4 mg; vitamin B_12_, 0.02 mg; pantothenate, 12 mg; niacin, 40 mg; folate, 1 mg; biotin, 0.02 mg*.

c *Provided per kg of diet (mineral): Zn, 90 mg; Mn, 100 mg; I, 1 mg; Cu, 15 mg; Fe, 90 mg; I, 200 mg; Se, 0.15 mg; Co, 0.25 mg*.

d *ME, metabolizable energy*.

The feeding strategy was adapted from the Broiler Management Manual that divided growth into different growth stages. The average body weight, average daily gain, average daily feed intake, and feed efficiency (total weight gain/total feed intake) were calculated from days 1 to 30. At the end of experiment, chickens were sacrificed with an anesthetic and stunned with carbon dioxide. All research was approved by the Tunghai University Institutional Animal Care and Use Committee (IACUC Approval No. 107-6) prior to the start of data collection.

### Histological Examination

On day 30, intestinal sample from 2 broilers per replicate were freshly collected. Four replicates (eight birds/treatment, *n* = 8) were used for histological examination. Intestinal proximal segment samples (~3 cm in length) of the duodenum, jejunum and ileum were excised and flushed with PBS to remove the residues. The intestinal samples were fixed in a 10% buffered formalin solution for histopathological examination. Tissues were embedded in paraffin wax blocks, sectioned at 5 μm thickness and stained with hematoxylin and eosin. The villus height was examined in eight samples per group and each sample was photographed in 6 different fields randomly.

### DNA Extraction and Microbiota Analysis

For microbiome analysis, cecal contents from broilers per replicated were freshly collected at the end of experiment. Five samples from each treatment were randomly selected and used for microbiota analysis. The DNA was extracted using a commercial kit (QIAamp Fast DNA Stool Mini Kit, QIAGEN, Hilden, Germany) following the instructions of the manufacturer. The quality and quantity of DNA was evaluated on a SIMPLINANOTM spectrophotometer (SimpliNano, 29061711). DNA samples were stored at −20°C until further processing.

The 300 bp paired-end raw reads derived from the 16S ribosomal amplicon sequencing were assembled using FLASH v.1.2.11 (29). De-multiplexing was carried out based on barcode identification (30). The raw reads were required to match the correct barcode and primer. Read pairs were then exported into dataset-specific, oriented files. Barcode files were generated at this step, and facilitate incorporation of the datasets into the QIIME. As a quality control, reads with a *Q* score less than the threshold (*Q* < 20) were discarded in the QIIME 1.9.1 pipeline (30). If three consecutive bases were *Q* < 20, the read was truncated and the resulting read retained in the data set only if it was at least 75% of the original length using split_libraries_fastq.py script in QIIME (31). Sequences were chimera-checked using UCHIME to obtain the effective tags (32, 33) and filtered from the data set before operational taxonomic unit (OTU) clustering at 97% sequence identity using the UPARSE function in the USEARCH v.7 pipeline (34). For each representative sequence, the RDP classifier (v.2.2) algorithm (35) was employed to annotate taxonomy classification based on the information retrieved from the Silva Database v.132 (36, 37). Sequences with one-time occurrence (singletons) or present in only one sample were filtered out.

Samples were grouped according to treatment. Analysis was performed at different taxonomical levels separately (phylum, family, and genus). For statistical analysis, significance of all species among groups at various taxonomic level were detected using differential abundance analysis with a zero-inflated Gussian (ZIG) log-normal model as implemented in the “fitFeatureModel” function of the Bioconductor metagenomeSeq package (38). Statistically significant biomarkers were identified by the use of the LEfSe analysis (39). In brief, LEfSe is an approach based on an algorithm that performs the non-parametric Kruskal-Wallis test to identify bacterial taxa whose relative abundance is significantly different between the control and sample of interest. LEfSe applies LDA to those bacterial taxa identified as significantly different and further assesses the effect size of each differentially abundant taxon. In this study, taxa with LDA score (log 10) > 4 was considered significant.

### ELISA and Blood Biochemical Values

At the end of experiment, plasma samples from two broilers per replicate (eight birds/treatment, *n* = 8) were collected from brachial vein and separated through centrifugation at 2,500 × g for 20 min. Plasma from different groups were used to measure the blood biochemistry parameters and inflammation-related cytokines. The samples were diluted with saline, and concentrations of glucose (GLU), triglyceride (TG), cholesterol (CHOL), high-density lipoprotein (HDL), low-density lipoprotein (LDL) were measured by biochemistry automatic analyzer (FUJI DRI-CHEM NX 500i, Fujifilm Co., Japan). Total concentration of plasma Interleukin-6 (IL-6) (E12I0006, Shanghai Blue Gene Biotech), and Interleukin (IL-1 β) (E12I0010, Shanghai Blue Gene Biotech) were measured by using an ELISA microplate reader (Epoch2^TM^, BioTek).

### Statistical Analysis

The Kolmogorov-Smirnov test was used to test the normal distribution of the data before statistical analysis was performed. Statistical analyses were performed using GraphPad software (version 5 for Windows). The collected data were tested by means of one-way ANOVA and the mean differences were compared using Tukey's multiple comparison test. Significance was declared at *P* ≤ 0.05.

## Results

### Growth Performance

During the entire experimental period, the broilers showed good survival rates (Table 2). Live weights, average daily gain, and feed efficiency did not differ for the different dietary treatments (Table 2).

**Table 2:** Dietary effects of feeding AMX, SP1, SP2, MBV-SP1, and MBV-SP2 on growth performance of broilers.

	**CON**	**AMX**	**SP1**	**SP2**	**MBV-SP1**	**MBV-SP2**	***P*-value**
**BW (g)**							
1 D	43.02 ± 0.04	43.05 ± 0.05	43.09 ± 0.04	43.10 ± 0.02	43.05 ± 0.04	43.02 ± 0.03	0.99
30 D	1630 ± 102.40	1640 ± 104.12	1528 ± 98.30	1499 ± 92.80	1490 ± 80.05	1565 ± 95.06	0.30
ADFI	67.06 ± 2.88	69.21 ± 3.02	64.48 ± 2.60	63.39 ± 3.70	64.27 ± 3.01	65.08 ± 3.31	0.29
FE	0.66 ± 0.02	0.69 ± 0.03	0.67 ± 0.03	0.63 ± 0.03	0.66 ± 0.04	0.67 ± 0.02	0.09
Survival %	96	100	100	98	100	100	-

*Data were presented as mean ± S.D. The means of each pens were used as the experimental units for all variables evaluated. Control (basal diet); AMX, basal diet plus 0.1% amoxicillin; SP1, basal diet plus 0.05% microalgae; SP2, basal diet plus 0.1% microalgae; MBV-SP1, basal diet plus 0.05% MBV-enriched microalgae; MBV-SP2, basal diet plus 0.1% MBV-enriched microalgae; BW, body weight; D, day; ADFI, average daily feed intake (g/D/bird); FE, feed efficiency; ADG, average daily gain (n = 4/each group)*.

### Histological Analysis

**Figure 1:**
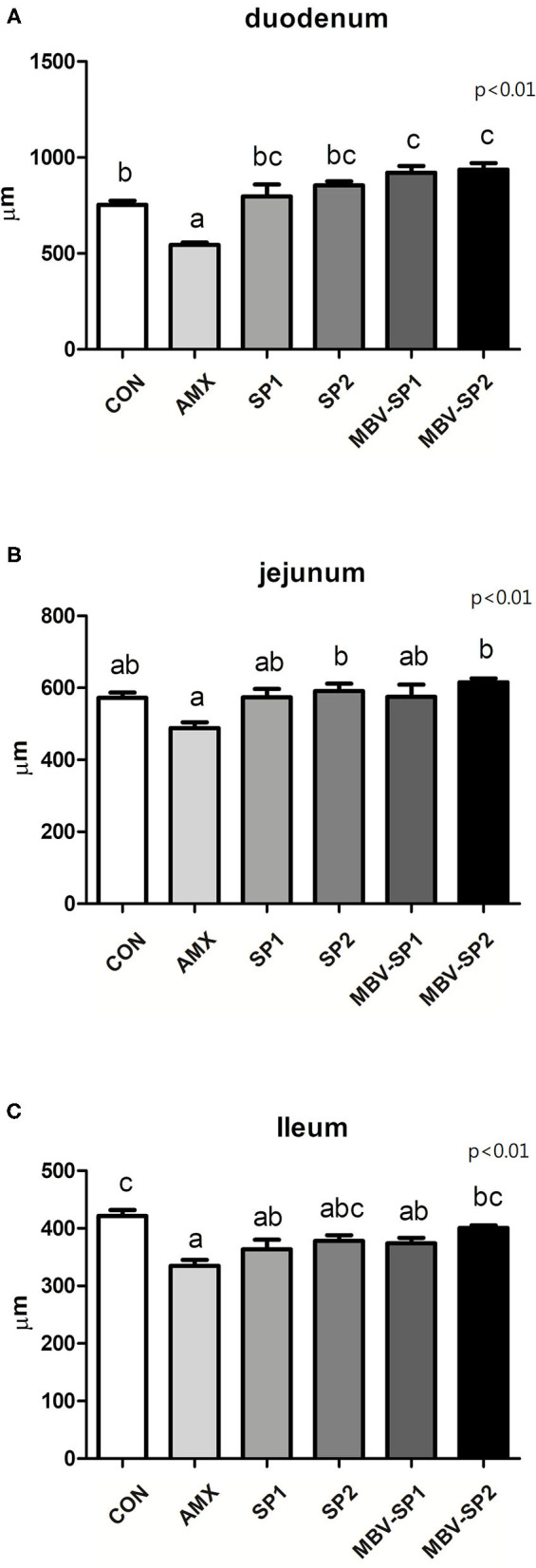
Histological analysis of broiler small intestine after feeding with basal diet (CON), AMX, SP1, SP2, MBV-SP1, and MBV-SP2 of **(A)** duodenum, **(B)** jejunum, and **(C)** ileum. Data were presented as mean ± S.D. and subjected to one-way ANOVA. Different letters indicate significant differences between treatments. Significance was declared when the probability was <5% (*P* < 0.05; *n* = 8/each group).

Histological analysis of intestinal segments showed that AMX treatment decreased villi lengths of the duodenum, jejunum, and ileum below those observed with control (Figure 1). In contrast, MBV-SP1 and MBV-SP2 treatments resulted in increased villi lengths in the duodenum above those observed with control. SP1, SP2, MBV-SP1, and MBV-SP2 treatments showed higher degrees of villi lengthening than observed for AMX treatments with MBV-SP2 showing the highest degree of increased villi lengths (*P* < 0.05; Figure 1). Representative histological images of chickens of different intestinal segment were presented in Supplementary Figure 1.

### Microbiota Analysis

The species accumulation curve (Supplementary Figure 2) indicates an increase in species diversity as the sample size increases. For investigating the microbiota population, the species accumulation curve can be used to assess whether the number of samples provided in the current sequence analysis is sufficient. If the curve rises sharply, a large number of species is found in the community, and when the curve flattens, the species number will not increase with a sample size increase. AMX treatment significantly increased the Firmicutes/Bacteriodetes (F/B) ratio (*P* < 0.05) while SP1 treatment had no significant impact on the F/B ratio (Supplementary Figure 3). However, SP2, MBV-SP1, and MBV-SP2 treatments increased the F/B ratio.

**Figure 2:**
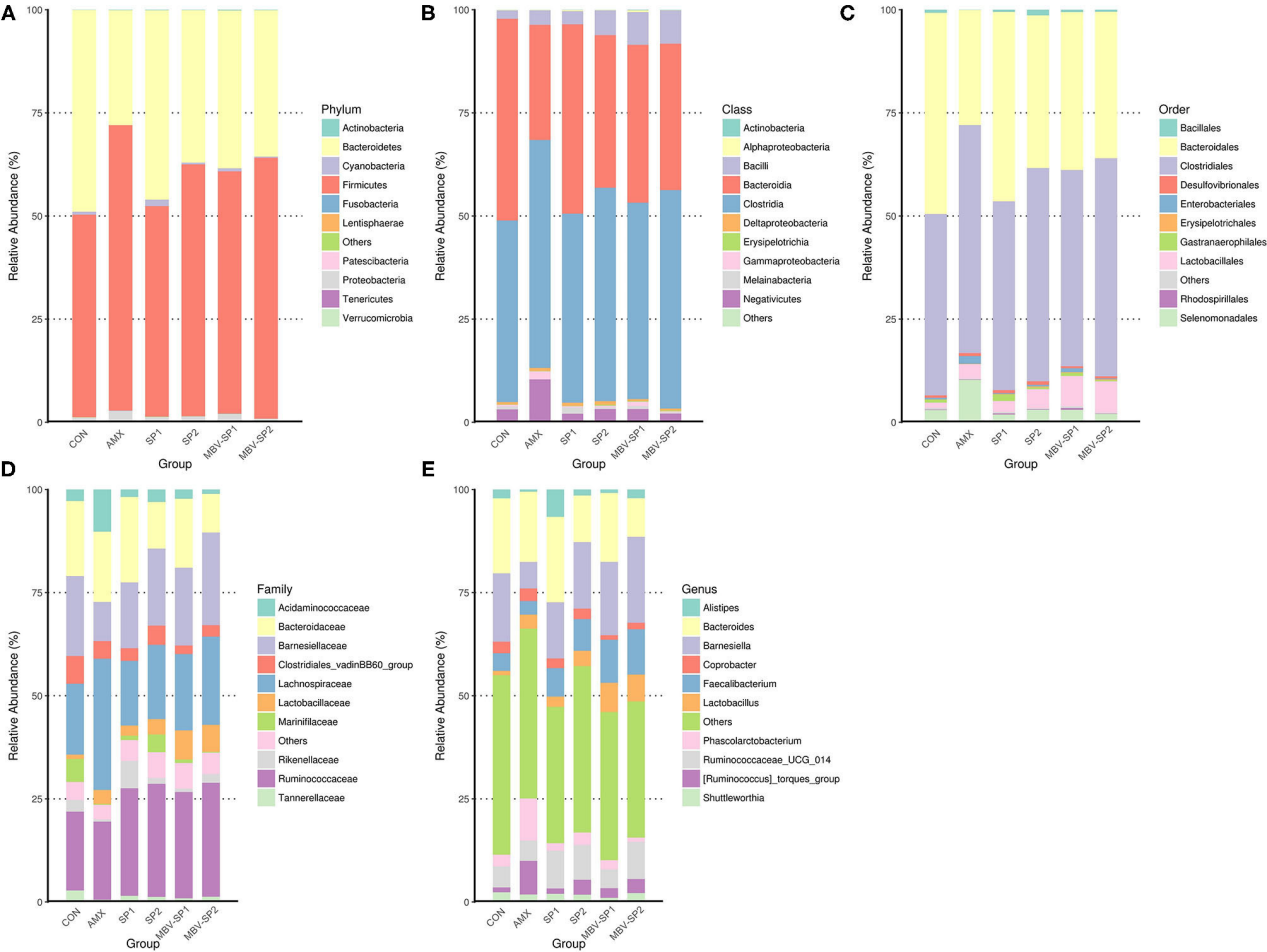
The relatively abundant microbiota in different taxonomic levels. **(A)** Phylum, **(B)** Class, **(C)** Order, **(D)** Family, and **(E)** Genus level. Each color represents a different taxonomic unit (*n* = 5/each group).

In order to explore the relative abundance of microbiota (phylum, class, order, family, and genus) we select the top 10 populations of species for relative abundance in each feed treatment group identified by the gene sequence analyses for species annotation. The relative proportions of different taxonomic classes were obtained for each feed treatment (Figure 2). The analyses show that *Firmicutes* and *Bacteroidetes* are the most common phyla in chicken ceca, and *Proteobacteria* and *Cyanobacteria* account for the remainder (Figure 2A). Changing the feed treatments significantly altered the F/B ratios (Supplementary Figure 3 and Figure 2A). AMX, MBV-SP1, and MBV-SP2 treatments increased the *Firmicutes* and reduced the *Bacteroidetes* populations. At the class level, the majority were *Clostridia, Bacteroidia, Bacilli*, and *Negativicutes* (Figure 2B), and at the order level, *Clostridiales, Bacteroidales, Lactobacillales*, and *Selenomonadales* comprised the majority (Figure 2C). At the family and genus levels, AMX, SP1, SP2, MBV-SP1, and MBV-SP2 each increased the proportions of *Lactobacillaceae* and *Lactobacillus*, respectively, with MBV-SP1 and MBV-SP2 having similar and the most dramatic effects (Figures 2D,E). Partial Least Squares Discriminant Analysis (PLS-DA) conducted to examine the functional distinction of microbiota revealed statistically significant discrimination among the groups (Supplementary Figure 4).

**Figure 3:**
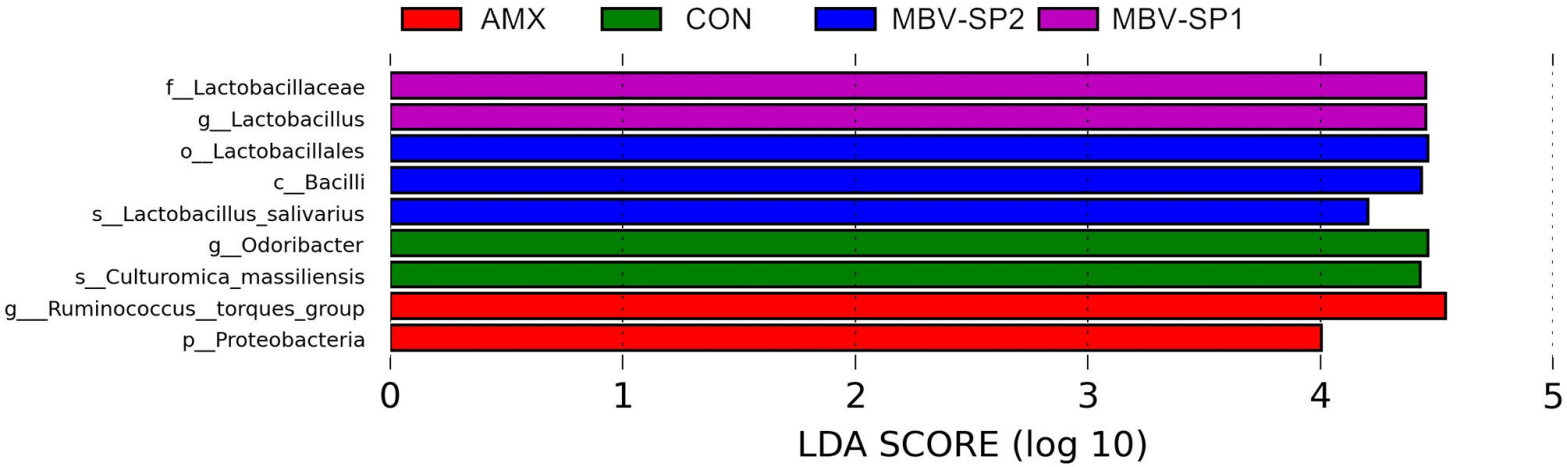
LEfSe analysis showing the most differentially abundant taxa between control and treatment groups. Only taxa with LDA > 4 are shown. The letter in front of the strains indicates the taxon level; p, phylum; c, class; o, order; f, family; g, genus; s, species (*n* = 5/each group).

**Figure 4:**
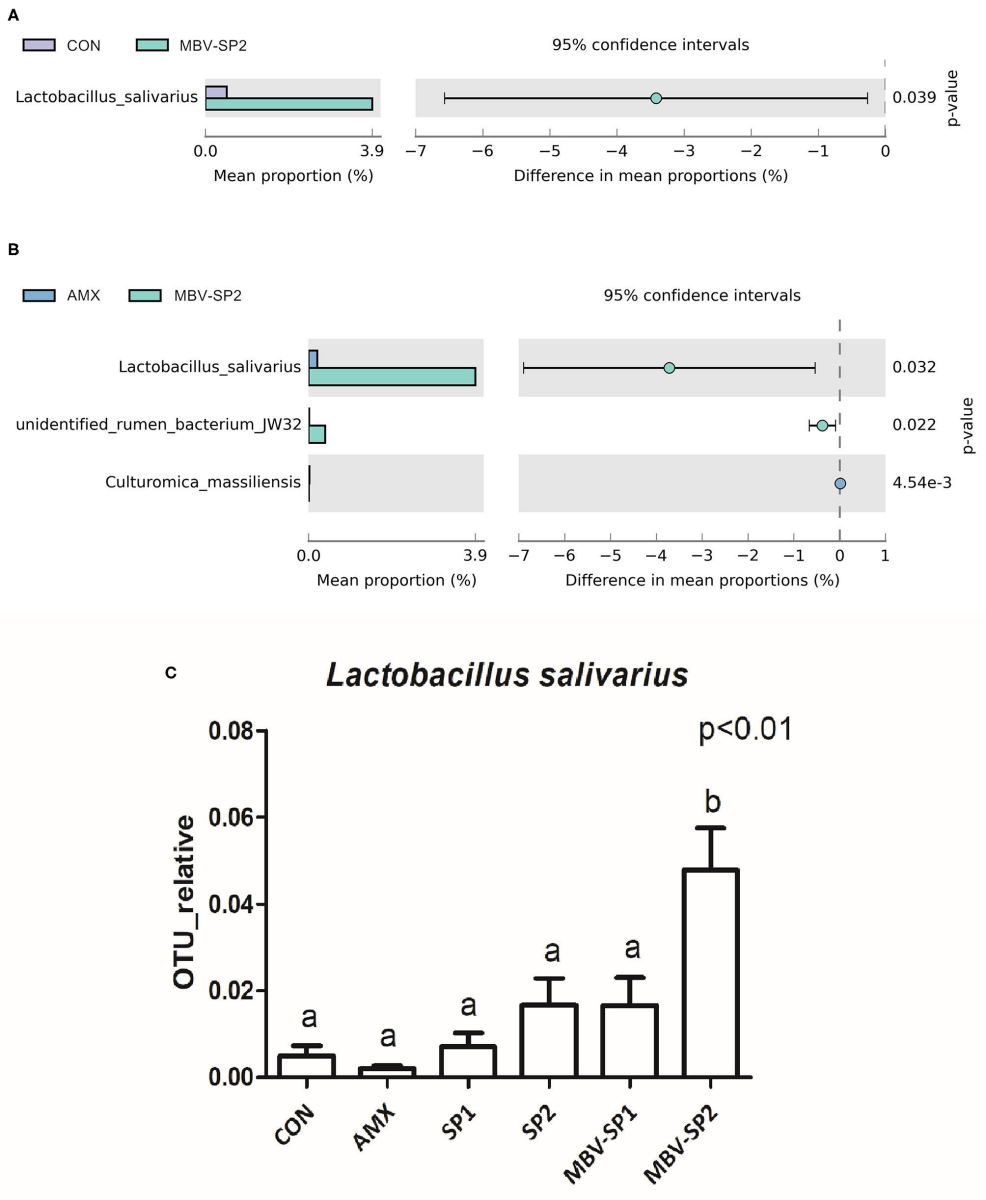
**(A)** The Welch's test of group CON and MBV-SP2, **(B)** The Welch's test of group AMX and MBV-SP2, **(C)** The OTU relative abundance of *Lactobacillus salivarius* after feeding with basal diet (CON), AMX, SP1, SP2, MBV-SP1, and MBV-SP2. Data were presented as mean ± S.D. and subjected to one-way ANOVA. The mean differences were compared using Tukey's multiple comparison test. Different letters indicate significant differences between treatments. Significance was declared when the probability was <5% (*P* < 0.05; *n* = 5/each group).

LEfSe applies LDA to those bacterial taxa identified as significantly different (*P* < 0.05) are presented as Figure 3. Statistical analyses of metagenomics profiles were used to perform Welch's *t*-test between pairs of feed treatment groups to determine which gut bacterial species displayed the largest difference in abundance when comparing two different feeding treatments (*P* < 0.05; Figures 4A,B). The analyses revealed that the proportion of *Lactobacillus salivarius* increased to the largest extent when comparing CON vs. MBV-SP2 (*P* < 0.05) and AMX vs. MBV-SP2 feeding treatments (*P* < 0.05). The calculated OTU relative abundance of *L. salivarius*, confirmed that MBV-SP2 is best among all treatments for increasing its abundance (Figure 4C).

### ELISA Analysis and Blood Biochemistry Parameters

**Figure 5:**
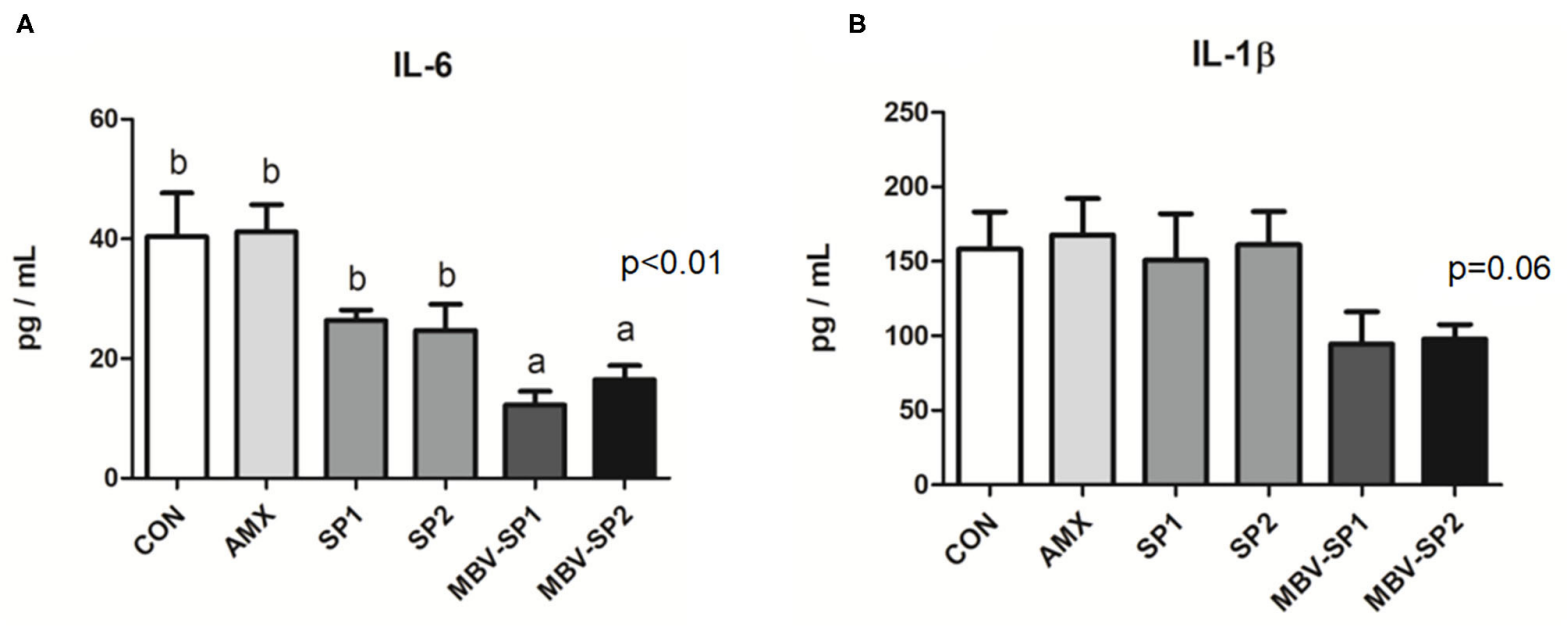
Enzyme-linked immunosorbent assay of IL-6 **(A)** and IL-1 beta **(B)** in chicken plasma after feeding with basal diet (CON), AMX, SP1, SP2, MBV-SP1, and MBV-SP2. Data were presented as mean ± S.D. and subjected to one-way ANOVA. The mean differences were compared using Tukey's multiple comparison test. Different letters indicate significant differences between treatments. Significance was declared when the probability was <5% (*P* < 0.05; *n* = 8/each group).

No significant differences between the blood biochemical values were observed for all of the feeding treatment and control groups (Table 3). It should be noted that a trend of decreased values for LDL (*P* = 0.1) levels was evident with MBV-SP2 treatment. Cytokine analysis revealed that MBV-SP1 and MBV-SP2 feeding treatment significantly decreased (*P* < 0.05) blood IL-6–an inflammatory biomarker in chickens–below the control and AMX treatments (Figure 5A). SP1 and SP2 feeding lowered IL-6 as well but to a lesser extent. Another prominent cytokine in chicken, IL-1β did not change levels with any of the feed treatments (Figure 5B). However, the downward trend can be observed in MBV-SP1 and MBV-SP2 feeding treatment.

**Table 3:** Effects of feeding AMX, SP1, SP2, MBV-SP1, and MBV-SP2 on blood biochemistry parameters in broilers.

**(mg/dl)**	**CON**	**AMX**	**SP1**	**SP2**	**MBV-SP1**	**MBV-SP2**	***P*-value**
GLU	301.13 ± 17.72	304.25 ± 18.67	317.75 ± 65.56	301.00 ± 25.15	314.50 ± 27.90	313.38 ± 41.21	0.89
TG	140.88 ± 22.18	158.13 ± 35.95	133.88 ± 20.65	141.00 ± 32.11	120.25 ± 60.80	151.38 ± 16.09	0.34
CHOL	107.50 ± 19.21	105.25 ± 16.79	105.00 ± 10.90	107.13 ± 11.98	107.50 ± 15.36	104.50 ± 15.10	0.99
HDL	76.00 ± 7.96	84.25 ± 10.50	85.88 ± 7.68	86.00 ± 9.81	82.13 ± 15.26	84.38 ± 6.00	0.36
LDL	43.50 ± 10.41	39.50 ± 9.02	42.25 ± 4.77	36.50 ± 3.07	38.00 ± 5.35	35.00 ± 2.93	0.10

*Data were presented as mean ± S.D. GLU, glucose; TG, triglyceride; CHOL, cholesterol; HDL, high-density lipoprotein; LDL, low-density lipoprotein. Control (basal diet); AMX, basal diet plus 0.1% amoxicillin; SP1, basal diet plus 0.05% microalgae; SP2, basal diet plus 0.1% microalgae; MBV-SP1, basal diet plus 0.05% MBV-enriched microalgae; MBV-SP2, basal diet plus 0.1% MBV-enriched microalgae (n = 8/each group)*.

## Discussion

Spirulina microalgae is not only used as a raw material for animal and aquatic feed, but it also has microbial-modulating activities, both for Gram-negative and Gram-positive bacteria (40–42). Moreover, spirulina also has beneficial effects on metabolic disease in clinical trial and alleviates adverse impacts due to high ambient temperature that reduces immunity function and increases production of free radicals in broilers (15, 43) which implies spirulina's potential as a nutraceutical in animals and humans. In the current study, we observe that spirulina extract enriched with MBV appears to possess even greater protective and beneficial effects for gut health than spirulina extract alone. This study suggests the possible use of MBV-enriched microalgae as a beneficial or medicinal animal feed or supplement.

Our study found that providing spirulina as feed supplement does not cause adverse growth effects (Table 2), and it alters the gut microbiota (Figures 2, 4). Our results are the first to show that microalgal spirulina extract containing MBV increases the abundance of *Lactobacillus*, especially *L. salivarius* in the gut of broilers (Figure 4C). Various studies have demonstrated that *L. salivarius* is a promising probiotic that produces bacteriocins inhibitory to the growth of other bacteria and occurs in human, porcine and poultry gastrointestinal tracts (44–47). Furthermore, *L. salivarius* has been shown to modulate inflammatory cytokines against critical gut pathogens *Salmonella* and *Campylobacter jejuni* (45, 46, 48). Our results are consistent with these previous findings and further show the improvement of intestinal structure and immune-modulating function by MBV-containing spirulina extract (Figures 1, 5). As for the mechanism underlying the effects of the adopted treatment, no previous published study on the use of MBV in animal feed exists. Biliverdin has been considered as a therapeutic agent for many years (19). However, considering its clinical application, there are insufficient quantities, uncertain purity, and obstacles derived from mammalian materials. As an alternative, our group synthesized MBV from microalgae phycocyanin with high purity and in large amounts. In the current study, we do not clarify the specific mechanisms underlying the effects of MBV-SP treatment. However, two important gut health parameters are correlated with the observed gut microbiota index changes: normal intestinal histopathology (Figure 1) and lowered plasma IL-6 and IL-1β levels (Figure 5) both known to be associated with suppression of intestinal bowel disease (49–52). Therefore, it is speculated that MBV improves gut health via modulation of gut microbiota and lowered circulatory inflammatory cytokines. In recent experiments using pig intestinal cells as a platform and purified MBV, we observed a cytoprotective effect (unpublished data) that is conceivably relevant to our findings reported here.

The current research examines the possibility of a new feed additive of livestock feed devoid of antibiotics and that could promote animal gut health. It exploits recent discoveries about MBV, an analog of the animal metabolite biliverdin IXα known to protect against inflammatory conditions such as IBDs. Future research is aimed at scaled production of MBV-enriched microalgae extract and determination of efficacy toward development of a next- generation animal feed.

## Conclusions

Our data reveal MBV-SP1 and particularly MBV-SP2 increased villi lengths in the duodenum, jejunum, and ileum above AMX-treatment lengths. In addition, feeding MBV-SP1 and MBV-SP2 decreased the level of pro-inflammatory cytokine IL-6 in plasma of broilers to a greater extent than SP1 and SP2. The microbiota analysis also showed that MBV-enriched microalgae spirulina have beneficial effects for gut health. In sum, MBV-enriched microalgae extracts may replace the antibiotics used in livestock industry.

## Data Availability Statement

The datasets presented in this study can be found in online repositories. The names of the repository/repositories and accession number(s) can be found below: European Bioinformatics Institute, accession no: PRJEB41302.

## Ethics Statement

The animal study was reviewed and approved by Tunghai University.

## Author Contributions

Y-YL and P-EC designed the research. C-WC, JT, and MA provide materials (microalgae extract and MBV-enriched microalgae extract). Y-YL and P-EC performed the research and analyzed the data. Y-YL wrote the manuscript. C-WC, JT, and Y-YL participated in the revision of the manuscript. All authors contributed to data interpretation and approved the final version of the manuscript.

## Conflict of Interest

The authors declare that the research was conducted in the absence of any commercial or financial relationships that could be construed as a potential conflict of interest.

## Supplementary Material

Supplemental Figure 1 Representative histological images of different intestinal segment. Sections were stained with hematoxylin and eosin and viewed at X40 magnification with scale bars indicating 500 μm.

Supplemental Figure 2 Species accumulation curve. Data were presented as mean ± S.D.

Supplemental Figure 3 Effect of SP1, SP2, MBV-SP1, and MBV-SP2 feed on broiler gut *Firmicutes*/*Bacteroidetes* (F/B) ratios. Data were presented as mean ± S.D.

Supplemental Figure 4 Partial Least Squares Discriminant Analysis of the cecal contents. Control (basal diet); AMX, basal diet plus 0.1% amoxicillin; SP1, basal diet plus 0.05% microalgae; SP2, basal diet plus 0.1% microalgae; MBV-SP1, basal diet plus 0.05% MBV-enriched microalgae; MBV-SP2, basal diet plus 0.1% MBV-enriched microalgae.
