# Supplementary figures and images for: Effects of Mesobiliverdin IXα-Enriched Microalgae Feed on Gut Health and Microbiota of Broilers
Source: Front Vet Sci. 2021 Jan 20;7:586813. doi: 10.3389/fvets.2020.586813 (PMC7854538; doi:10.3389/fvets.2020.586813)

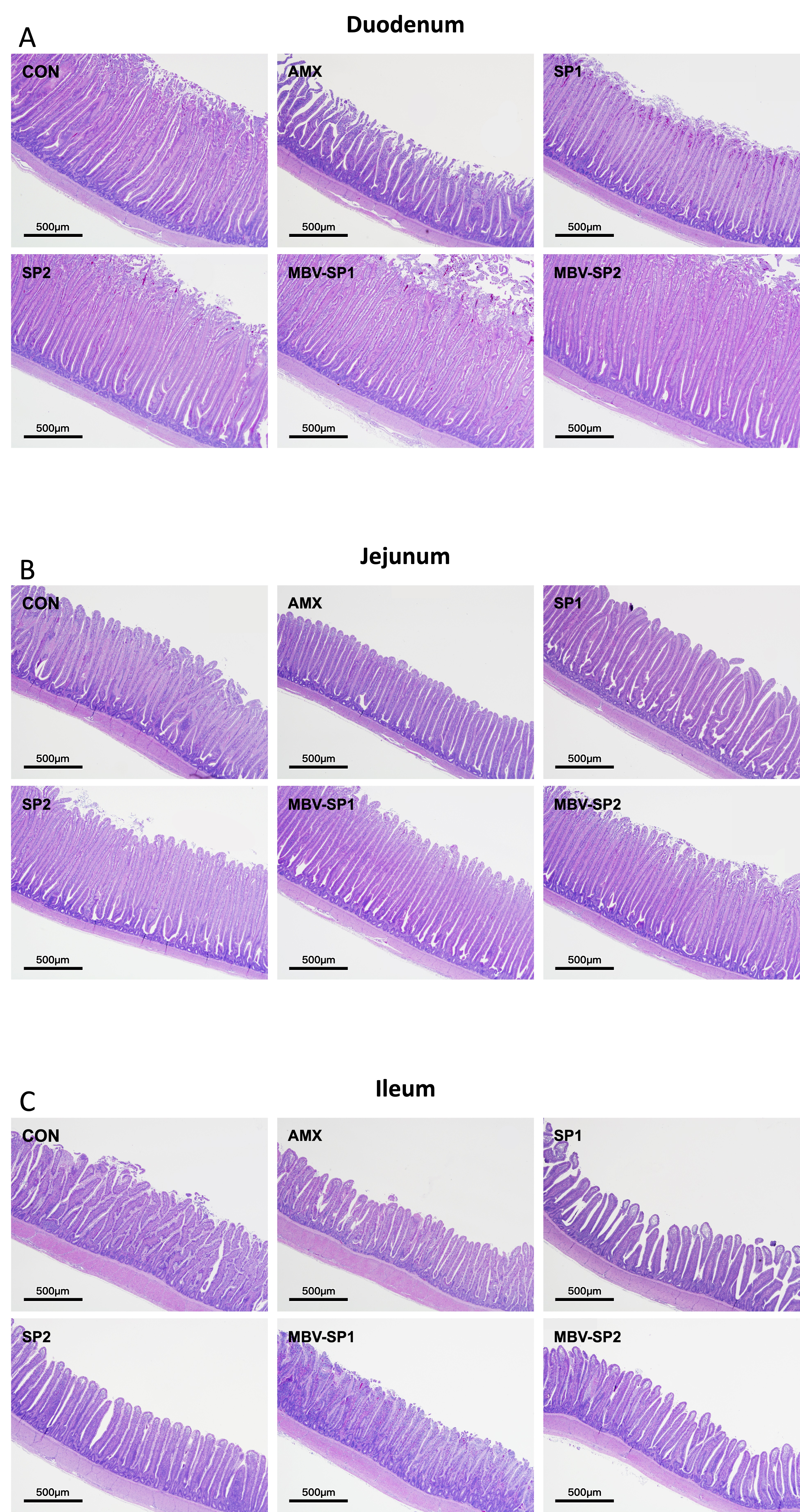

Supplement: Supplemental Figure 1 — Representative histological images of different intestinal segment. Sections were stained with hematoxylin and eosin and viewed at X40 magnification with scale bars indicating 500 μm. [file Image_1.JPEG]

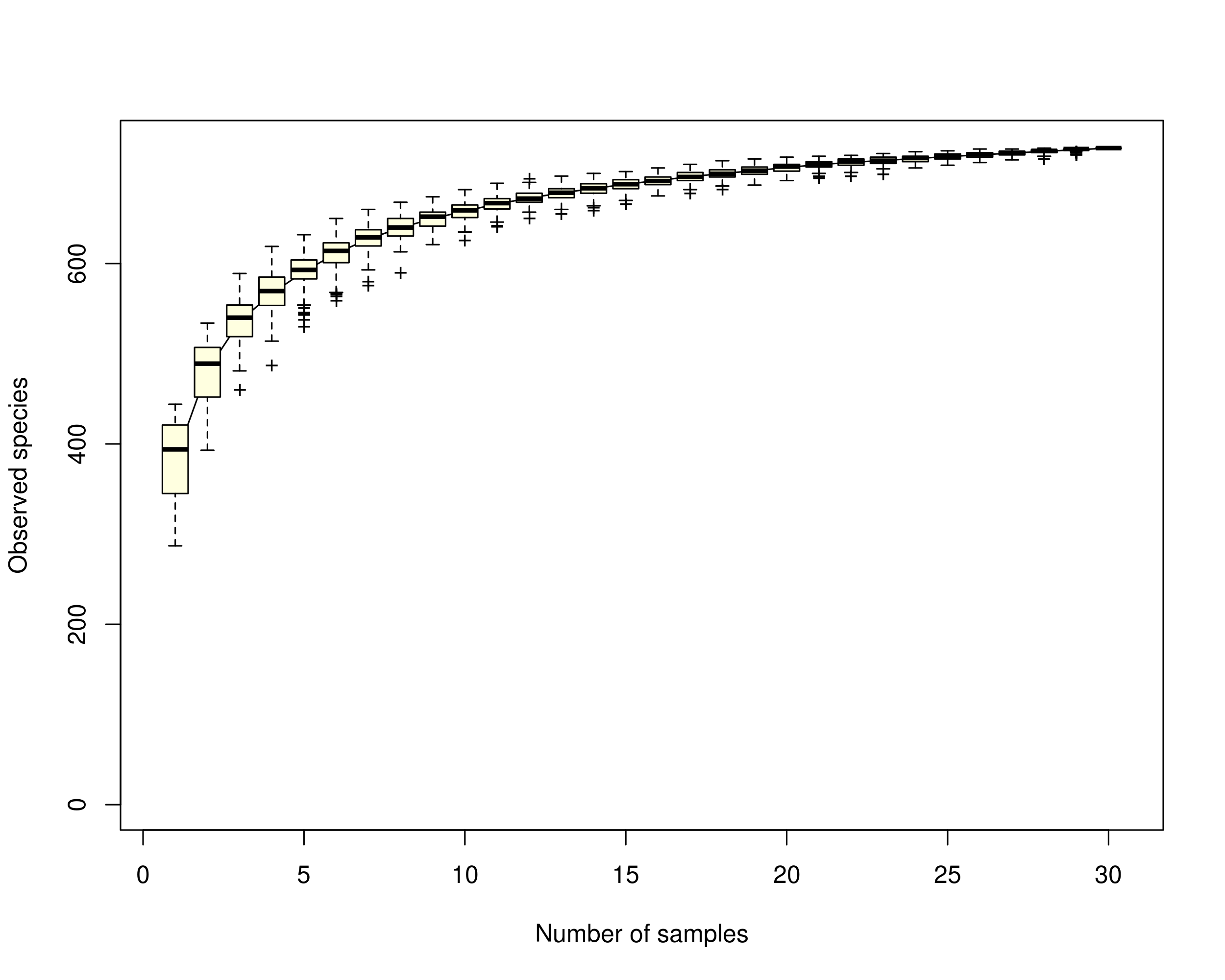

Supplement: Supplemental Figure 2 — Species accumulation curve. Data were presented as mean ± S.D. [file Image_2.PNG]

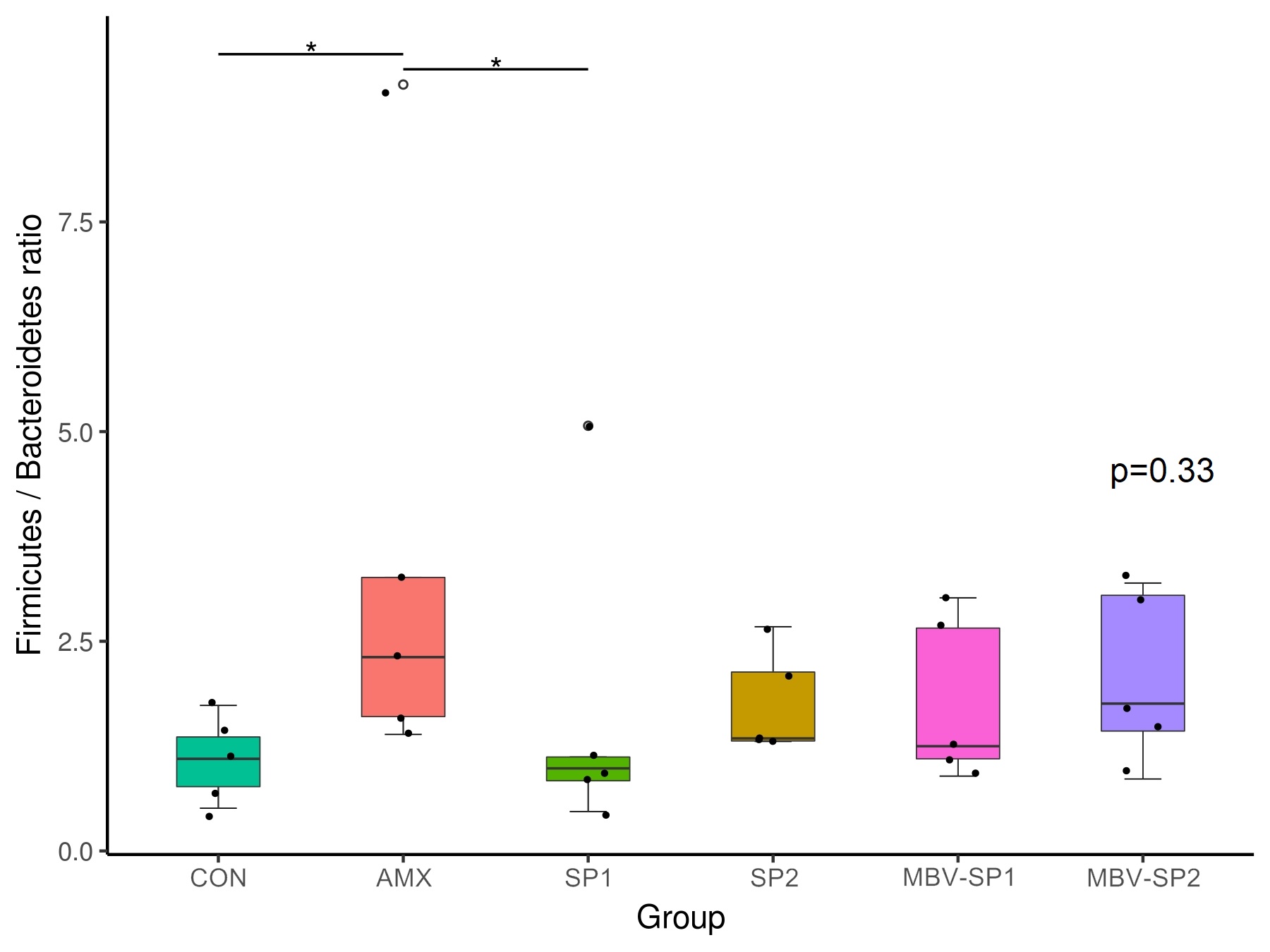

Supplement: Supplemental Figure 3 — Effect of SP1, SP2, MBV-SP1, and MBV-SP2 feed on broiler gut Firmicutes/Bacteroidetes (F/B) ratios. Data were presented as mean ± S.D. [file Image_3.JPEG]

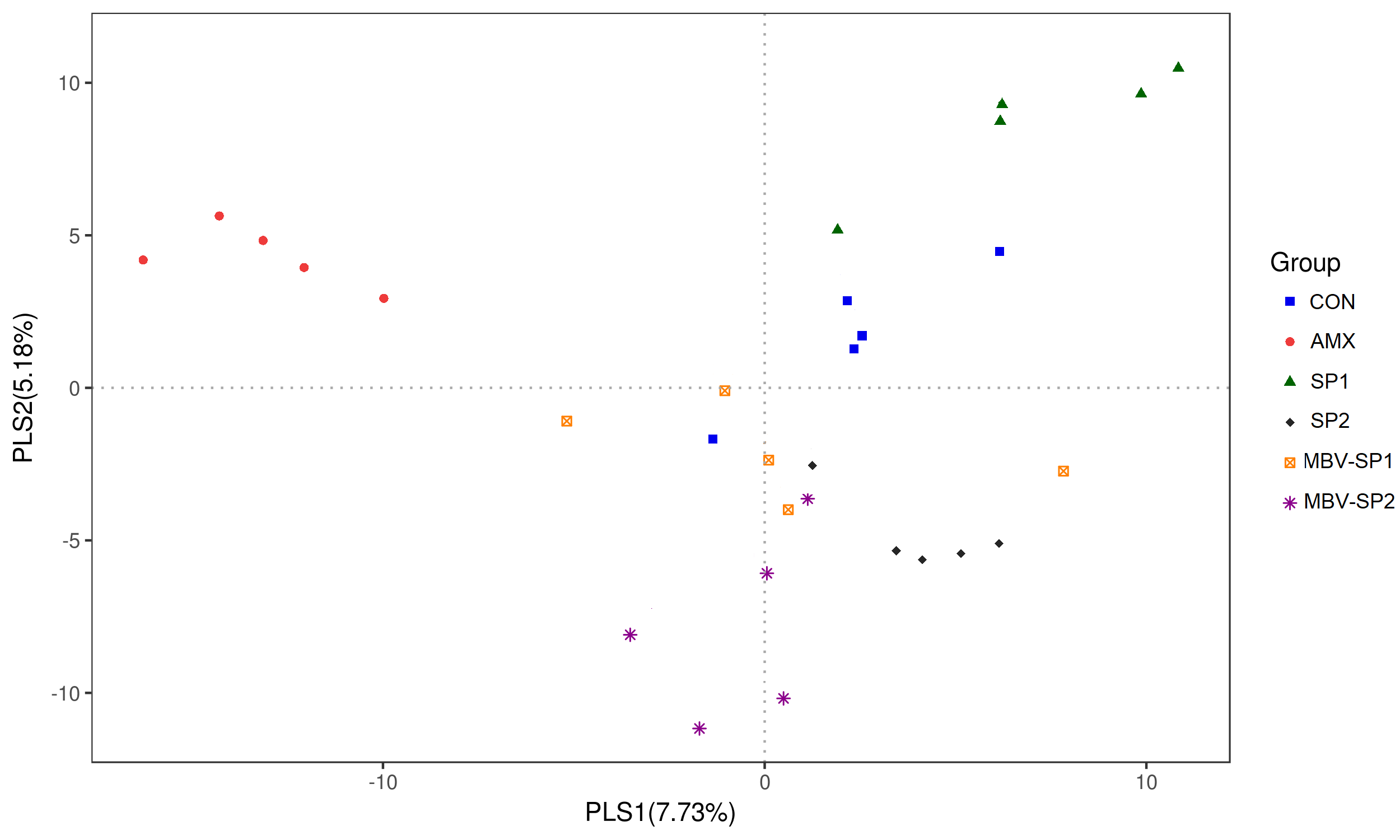

Supplement: Supplemental Figure 4 — Partial Least Squares Discriminant Analysis of the cecal contents. Control (basal diet); AMX, basal diet plus 0.1% amoxicillin; SP1, basal diet plus 0.05% microalgae; SP2, basal diet plus 0.1% microalgae; MBV-SP1, basal diet plus 0.05% MBV-enriched microalgae; MBV-SP2, basal diet plus 0.1% MBV-enriched microalgae. [file Image_4.PNG]
